# Borrowing strength from clinical trials in analysing longitudinal data from a treated cohort: investigating the effectiveness of acetylcholinesterase inhibitors in the management of dementia

**DOI:** 10.1093/ije/dyac185

**Published:** 2022-10-11

**Authors:** Ruth Knight, Robert Stewart, Mizanur Khondoker, Sabine Landau

**Affiliations:** Oxford Clinical Trials Research Unit, Centre for Statistics in Medicine, University of Oxford, Oxford, UK; Department of Biostatistics and Health Informatics, Institute of Psychiatry, Psychology and Neuroscience, King’s College London, London, UK; Department of Psychological Medicine, Institute of Psychiatry, Psychology and Neuroscience, King's College London, London, UK; South London and Maudsley NHS Foundation Trust, London, UK; Norwich Medical School, University of East Anglia, Norwich, UK; Department of Biostatistics and Health Informatics, Institute of Psychiatry, Psychology and Neuroscience, King’s College London, London, UK

**Keywords:** Randomized controlled trial, electronic medical record, Bayesian modelling, dementia, cognition, acetylcholinesterase inhibitors

## Abstract

**Background:**

Health care professionals seek information about effectiveness of treatments in patients who would be offered them in routine clinical practice. Electronic medical records (EMRs) and randomized controlled trials (RCTs) can both provide data on treatment effects; however, each data source has limitations when considered in isolation.

**Methods:**

A novel modelling methodology which incorporates RCT estimates in the analysis of EMR data via informative prior distributions is proposed. A Bayesian mixed modelling approach is used to model outcome trajectories among patients in the EMR dataset receiving the treatment of interest. This model incorporates an estimate of treatment effect based on a meta-analysis of RCTs as an informative prior distribution. This provides a combined estimate of treatment effect based on both data sources.

**Results:**

The superior performance of the novel combined estimator is demonstrated via a simulation study. The new approach is applied to estimate the effectiveness at 12 months after treatment initiation of acetylcholinesterase inhibitors in the management of the cognitive symptoms of dementia in terms of Mini-Mental State Examination scores. This demonstrated that estimates based on either trials data only (1.10, SE = 0.316) or cohort data only (1.56, SE = 0.240) overestimated this compared with the estimate using data from both sources (0.86, SE = 0.327).

**Conclusions:**

It is possible to combine data from EMRs and RCTs in order to provide better estimates of treatment effectiveness.

Key Messages

- Data on a treated cohort from an electronic medical record (EMR) and data from randomized controlled trials (RCTs) can be combined to provide estimates of treatment effects that are less biased and more generalizable than those from either data source alone.
- This holds true even if both are biased in the same direction.
- Estimates from either EMRs or RCTs alone overestimate the effects of acetylcholinesterase inhibitors in terms of Mini-Mental State Examination scores at 12 months after treatment initiation.
- It is possible to combine data from observational and randomized data sources even when the observational data are not comparative.
- A concerted effort to assemble routine EMR data in a form that can be used to improve real-world inferences from RCTs is required.

## Introduction

Health care professionals seek knowledge of the effectiveness of treatments in patients who would be offered them in routine clinical practice. Electronic medical records (EMRs) provide potentially valuable representative longitudinal data on treatment outcomes in routine clinical practice.^1^^,^^2^ However, the absence of an adequate control group can often limit estimates of treatment effects. On the other hand, randomized controlled trials (RCTs) should provide an unbiased estimate of treatment effect in the population in which they are conducted,^3^ but may lack generalizability to patients who will be given the treatment in routine practice.^4^^,^^5^ Combining data from both sources may help provide estimates that are both unbiased and generalizable.

Development of methods to combine data from both randomized and observational data sources is an ongoing area of research, with a variety of methods developed in recent years.^6–8^ An early, influential approach was the confidence profile method,^9^ a direct application of Bayesian modelling which emphasizes a case-specific modelling approach. Meta-analysis is popular (e.g. Prevost et al.,^10^, Larose and Dey^11^); however, these methods tend to combine aggregate-level data only and require comparative data from both sources. Similarly, using a Bayesian model to incorporate aggregate-level data from one source as an informative prior distribution when analysing the other,^12^^,^^13^ also requires comparative data from both sources. Cross-design synthesis^14^^,^^15^ combines individual-level data from observational studies with aggregate-level data from RCTs, and involves the adjustment of individual study results for biases, followed by the combination of results within and across designs. The clinical applications of such methods have been limited, due to methodological complexity and individual-level data requirements. There is need for further research in this area, particularly in regard to methods that use EMR data which are a growing source of information. Many of the existing methods depend on having comparative data from an observational study rather than cohort data from an EMR.

In this paper, we propose a novel methodology for combined modelling which provides an estimator of treatment effectiveness which overcomes both the lack of an adequate control group in EMR data and the lack of generalizability in RCT data. A Bayesian approach combines these data sources, incorporating RCT estimates as part of an informative prior distribution.

The motivating clinical question for this work is the estimation of the effectiveness of acetylcholinesterase inhibitors (AChEIs) in managing the cognitive symptoms of dementia. Dementia is a major health concern, affecting 47 million sufferers worldwide in 2016, predicted to rise to 131 million by 2050.^16^ There is currently no cure for most forms of dementia; however, AChEIs are often prescribed to manage cognitive symptoms.^17^ These drugs have been prescribed in routine clinical practice for several years, and one source of pseudonymized data on their use is the South London and Maudsley Biomedical Research Centre case register.^18^ This EMR has been used to provide follow-up on a treated cohort of patients with a wide variety of comorbidities,who receive a range of concurrent medications.^19^ The most commonly applied measure of cognition used in routine dementia assessment and care is the Mini-Mental State Examination (MMSE^20^), generating scores ranging from 0 to 30 with higher scores indicating better cognition. There can be situations where a patient is not able to complete all items of the MMSE for reasons unrelated to their cognition (e.g. vision impairment, mobility restrictions etc). In this case, the score may be expressed as being out of a different total (e.g. 24/29). In the remainder of this paper we will refer to the number of questions asked of a patient as the denominator and the number answered correctly as the numerator. The effects of AChEIs have also been investigated in a large number of RCTs, and we recently conducted a systematic review and meta-analysis of these data.^21^ Synthesis of both sources of evidence offers the promise of a better estimate of the effectiveness of these treatments in routine clinical practice.

## Methods

### Description of data

The treated cohort used in this study was extracted from the South London and Maudsley Biomedical Research Centre case register. Patients were included in the cohort if: (i) they had at least one mention of an AChEI (donepezil, galantamine, rivastigmine) for which the date of treatment offer (approximated by treatment start date which is coded as the earliest date on any AChEI prescription) could be identified; (ii) they had at least one MMSE score with a denominator ≥24 recorded between 1 year before and 3 years after treatment offer (only a single MMSE score was required for inclusion and this could be before or after treatment initiation); and (iii) they had received a primary or secondary diagnosis of dementia excluding diagnoses of Parkinson’s disease dementia and dementia with Lewy bodies. For each eligible patient, all MMSE scores recorded between 1 year before and 3 years after treatment were extracted. MMSE scores with a denominator less than 30 were standardized by calculating an adjusted score as numerator divided by the denominator multiplied by 30. The treated cohort contained 3134 patients with a total of 13 577 scores between them, and covered the period 1 January 2005 to 8 February 2015. A previous systematic review and meta-analysis of trials of AChEIs in managing the cognitive symptoms of dementia forms the RCT dataset.^21^

### Estimator of treatment effect based on treated cohort alone

Each member of the target population, that is patients who receive this treatment in routine clinical practice, can be thought of as having two potential outcome trajectories: the one they would have followed if they were offered treatment, and the one they would have followed if they were not. In practice, only the first of these is observed. Using t to denote time, with time 0 being the point of treatment offer, these two outcomes can be summarized as: where y_ij, R=1_ is the outcome for individual i at time t_ij_ if they are offered treatment, and y_ij, R=0_ is the outcome for individual i at time t_ij_ if they are not offered treatment. For an individual, the effect of treatment offer (Δ_i_) at a fixed time t = α > 0, is the difference between their outcome at α if they were and were not offered treatment:


$$y_{\text{ij},\;R=1}|{\;t}_{\text{ij}}∼N\left(\mu_{1}\left(t_{\text{ij}}\right),\;\sigma^{2}\right)\;\text{if the participant was offered treatment}$$

(1) $$y_{\text{ij},\;R=0}|{\;t}_{\text{ij}}∼N\left(\mu_{0}\left(t_{\text{ij}}\right),\;\sigma^{2}\right)\;\text{if the participant was not offered treatment}$$

(2) $$\Delta_{i}\left(\alpha \right)={\;y}_{\text{ij},\;R=1}\left|\alpha \;–{\;y}_{\text{ij},\;R=0}\right|\alpha$$

For an individual it is possible to observe only one of the two potential outcomes; therefore, the parameter we are interested in estimating is the average effect of treatment offer at t = α, which we call the ARE (since it seeks to approximate the effect of randomized treatment allocation analysed under the intention-to-treat principle):


(3) $$\text{ARE}\left(\alpha \right)=E_{i}\left(\Delta_{i}\left(\alpha \right)\right)=E_{i}\left(y_{\text{ij}}{,}_{R=1}\left|\alpha \;–{\;y}_{\text{ij},\;R=0}\right|\alpha \right)=E_{i}\left(y_{\text{ij}}{,}_{R=1}|\alpha \right)\;–{\;E}_{i}\left(y_{\text{ij},\;R=0}|\alpha \right)=\mu_{1}\left(\alpha \right)\;–\;\mu_{0}\left(\alpha \right)$$

**Figure 1:**
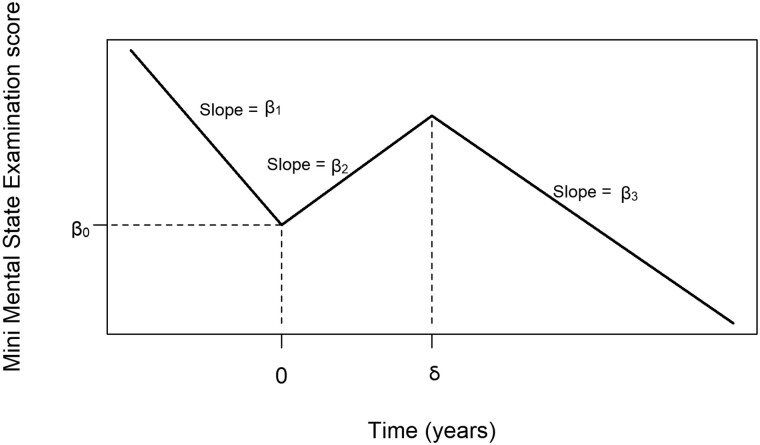
Piecewise linear model for Mini Mental State Examination trajectories

In order to be able to estimate this parameter, appropriate expressions for the average trajectory in the population who are offered treatment, and the population who are not, are needed. Previous work^22^ and non-parametric modelling of the current treated cohort have indicated that a piecewise linear mixed effects model (or alternatively linear spline) with two change points (or knot points), at treatment offer (t = 0) and at some subsequent unspecified time (t = δ >0), is appropriate to model the trajectory in those who are offered treatment (see Figure 1):


(4) $$\begin{array}{l} \mu_{1}\left(t\right)=\;\beta_{0}+\beta_{1}t,\;t<0\\ \;\;\;\;\;\;\;\;\;\;\;\;\;\;\;\;\;\;\;\;\;\beta_{0}+\beta_{2}t,\;0\leq t<\delta \\ \;\;\;\;\;\;\;\;\;\;\;\;\;\;\;\;\;\;\;\;\;\beta_{0}+\left(\beta_{2}-\beta_{3}\right)\delta +\beta_{3}t,\;t\geq \delta \\ \mu_{1}\left(t\right)=\;\beta_{0}+\beta_{1}{t\;1}_{t<0}+\beta_{2}\text{min}\left(t,\delta \right){\;1}_{t\geq 0\;}+\beta_{3}\left(t-\delta \right){\;1}_{t\geq \delta } \end{array}$$

All participants in the cohort were offered treatment, and so an assumption must be made about what would have happened had they not been offered treatment. The assumption made is that they would have continued on their pre-treatment trajectory (A1):


(5) $$\mu_{0}\left(t\right)=\beta_{0}+\beta_{1}t$$

Having made this assumption, it is possible to derive an expression for an estimator of treatment effect parameter (ARE). This estimator of treatment effect is denoted θ_α_, and may suffer from bias since it relies on assumption A1:


(6) $$\begin{array}{ll} \theta_{\alpha }=\mu_{1}\left(\alpha \right)\;–\;\mu_{0}\left(\alpha \right)=\left(\beta_{2}–\;\beta_{1}\right)\;\alpha , & \alpha <\delta \\ \left(\beta_{2}–\;\beta_{3}\right)\delta +\left(\beta_{3}–\;\beta_{1}\right)\alpha , & \alpha \geq \delta \end{array}\;$$

Equation (6) can be rearranged to express β_2_ in terms of the other parameters:


(7) $$\begin{array}{l} \beta_{2}=\frac{1}{\alpha }\theta_{\alpha }+\beta_{1,}\;\;\;\;\;\;\;\;\;\;\;\;\;\alpha <\delta \\ \frac{1}{\delta }\left[\theta_{\alpha }–\;\left(\beta_{3}-\beta_{1}\right)\alpha \right]+\beta_{3},\;\;\;\;\;\;\;\alpha \geq \delta \\ \beta_{2}=\beta_{1}(1_{\alpha <\delta }+\frac{\alpha }{\delta }\;1_{\alpha \geq \delta })+\theta_{\alpha }(\frac{1}{\alpha }1_{\alpha <\delta }+\frac{1}{\delta }1_{\alpha \geq \delta })+\beta_{3}(1-\frac{\alpha }{\delta })1_{\alpha \geq \delta } \end{array}$$

This expression for β_2_ can be substituted into the expected treated trajectory for those who are offered treatment [Equation (4)]:


(8) $$\begin{array}{l} \mu_{1}\left(t\right)=\beta_{0}+\beta_{1}({t\;1}_{t<0}+(1_{\alpha <\delta }+\frac{\alpha }{\delta }1_{\alpha \geq \delta })\;\text{min}\left(t,\delta \right){\;1}_{t>0})\\ +\;\theta_{\alpha }(\frac{1}{\alpha }1_{\alpha <\delta }+\frac{1}{\delta }1_{\alpha \geq \delta })\;\text{min}\left(t,\;\delta \right){\;1}_{t>0}\\ +\;\beta_{3}[(1-\frac{\alpha }{\delta })1_{\alpha \geq \delta \;}\text{min}\left(t,\delta \right){\;1}_{t>0}+\left(t-\delta \right)1_{t\geq \delta }] \end{array}$$

This model can be used to estimate the effect of treatment at time t = α (θ_α_) based on data from a cohort who were all offered treatment. Random effects on the coefficients can be incorporated to allow variation between patients:


(9) $$\begin{array}{l} \mu_{1}\left(t\right)=(\beta_{0}+b_{0i})+\left(\beta_{1}+b_{1i}\right)\\ \left({t\;1}_{t<0}+(1_{\alpha <\delta }+\frac{\alpha }{\delta }1_{\alpha \geq \delta })\;\text{min}\left(t,\delta \right){\;1}_{t>0}\right)\\ +\;\left(\theta_{\alpha }+{\;b}_{2i}\right)(\frac{1}{\alpha }1_{\alpha <\delta }+\frac{1}{\delta }1_{\alpha \geq \delta })\;\text{min}\left(t,\;\delta \right){\;1}_{t>0}+\\ \left(\beta_{3}+{\;b}_{3i}\right)[(1-\frac{\alpha }{\delta })1_{\alpha \geq \delta }\text{min}\left(t,\delta \right){\;1}_{t>0}+\left(t-\delta \right)1_{t\geq \delta }] \end{array}$$

To fit this model under a Bayesian framework, prior distributions for each of the parameters were determined. In the absence of additional information, non-informative priors should be used.^23^ For the coefficients, a suitable choice is a normal distribution with zero mean and large deviation. For the residual standard deviation, a suitable choice is uniform on the range 0–100. A suitable prior distribution for a change point parameter, such as δ, is a uniform prior on the range of possible values.^24^ In this instance, a plausible range is from 0 to 3, since the second change point must come after the first at t = 0 and the cohort consists of scores from 0 to 3 years after treatment offer. Suitable vague hierarchical priors are also placed on the random effects. For a single random effect, this is a normal distribution with mean 0 and variance σ_0_^2^ which is given a vague prior [U(0,100)]. In the presence of two or more random effects, these can be modelled using a multivariate normal distribution with mean zero. Vague priors are used for the covariance matrix. In the case of two random effects, the constituent parts of the covariance matrix can be given vague priors [U(0,100) for standard deviations and U(-1,1) for the correlation]. In the presence of three or more random effects, an inverse Wishart prior distribution is used for the re-scaled covariance matrix with U(0,100) priors used for the scaling parameters.^23^

### Incorporating RCT data via informative prior distributions

The assumption on which the estimator θ_α_ is based may be biased, patients may not have continued on their pre-treatment trajectory. This is called projection bias, with the projection bias at time = α denoted φ_α_. The true treatment effect, ARE(α), can be calculated as:


(10) $$\begin{array}{l} \text{ARE}\left(\alpha \right)=\theta_{\alpha }–\;\varphi_{\alpha }\\ \theta_{\alpha }=\text{ARE}\left(\alpha \right)+\varphi_{\alpha } \end{array}$$

This can be substituted into Equation (9) to give an expression for the MMSE trajectory in the treated cohort based on the true treatment effect and the projection bias, both at time = α:


(11) $$\begin{array}{l} \mu_{1}\left(t\right)=(\beta_{0}+b_{0i})+\left(\beta_{1}+b_{1i}\right)\\ \left({t\;1}_{t<0}+(1_{\alpha <\delta }+\frac{\alpha }{\delta }1_{\alpha \geq \delta })\;\text{min}\left(t,\delta \right){\;1}_{t>0}\right)\\ +\;(\text{ARE}\left(\alpha \right)+\varphi_{\alpha }+{\;b}_{2i})(\frac{1}{\alpha }\;1_{\alpha <\delta }+\frac{1}{\delta }\;1_{\alpha \geq \delta })\;\text{min}\left(t,\;\delta \right){\;1}_{t>0}\\ +\;(\beta_{3}+{\;b}_{3i})[(1-\frac{\alpha }{\delta })1_{\alpha \geq \delta }\text{min}\left(t,\delta \right){\;1}_{t>0}+\left(t-\delta \right)1_{t\geq \delta }] \end{array}$$

Data from RCTs can form the basis of an informative prior distribution for ARE(α); however, this is only true for the proportion of the target population who are trial eligible. The model in Equation (11) can be expanded to incorporate not only the possibility of different treatment effects in the trial-eligible and trial not-eligible populations, but also different trajectories within these two populations through the use of *S_i_*, which takes value 1 if individual i is trial eligible and 0 otherwise, to denote whether or not the individual is part of the trial-eligible population: where subscript 1 denotes parameters referring to the trial-eligible portion of the target population and subscript 2 denotes those for the trial not-eligible portion. The trial eligibility parameter is given a Bernoulli distribution: where 0 < π  <  1 is the proportion of the target population that are trial eligible. The overall treatment effect can be calculated as:


(12) $$\begin{array}{l} \mu_{1}\left(t\right)=(\beta_{01}1_{Si}{}_{=1}+\beta_{02}1_{Si=0}+b_{0i})\\ +\;\left(\beta_{11}1_{Si}{}_{=1}+\beta_{12}1_{Si}{}_{=0}+{\;b}_{1i}\right)\\ \left({t\;1}_{t<0}+(1_{\alpha <\delta }+\frac{\alpha }{\delta }1_{\alpha \geq \delta })\;\text{min}\left(t,\delta \right){\;1}_{t>0}\right)\\ +\;({\text{ARE}}_{1}\left(\alpha \right){\;1}_{Si}{}_{=1}+{\text{ARE}}_{2}\left(\alpha \right){\;1}_{Si}{}_{=0}+\varphi_{\alpha }+{\;b}_{2i})\\ (\frac{1}{\alpha }1_{\alpha <\delta }+\frac{1}{\delta }1_{\alpha \geq \delta })\;\text{min}\left(t,\;\delta \right){\;1}_{t>0}+\\ \left(\beta_{31}1_{Si}{}_{=1}+\beta_{32}1_{Si}{}_{=0}+b_{3i}\right)\\ \left[(1-\frac{\alpha }{\delta })1_{\alpha \geq \delta }\text{min}\left(t,\delta \right)1_{t>0}+\left(t-\delta \right)1_{t\geq \delta }\right] \end{array}$$

(13) $$S_{i}∼\;\text{Bin}\left(1,\;\pi \right)$$

(14) $$\text{ARE}\left(\alpha \right)=\pi {\text{ARE}}_{1}\left(\alpha \right)+(1-\pi ){\;\text{ARE}}_{2}\left(\alpha \right)$$

As before, each of the parameters in the model is given a prior distribution. An informative prior distribution^25^ based on meta-analysis of RCTs is used for ARE_1_(α). This meta-analysis was performed based on trials identified during a systematic review of the use of AChEIs in the management of dementia.^21^ Two steps were followed to convert the meta-analysis results to a suitable informative prior distribution^26^: (i) choosing an appropriate distribution; and (ii) using available information from the meta-analysis to provide estimates for the mean and variance. A normal distribution was selected with mean set as the pooled effect estimate from the meta-analysis and standard deviation set as the associated standard error. For other parameters, vague priors as described previously were used. Projection bias, φ_α_, can be both positive and negative and so was given a normal prior distribution with mean 0 and large variance. The trial-eligible proportion, π, is a probability and was thus given a uniform prior on the range 0 to 1.

This proposed combined model relies on two assumptions; first, that there are no treatment effect moderators whose distribution differs between the trial-eligible portion of the target population and the trial samples (A3); and second, that the projection bias, φ_α_, is the same in the trial-eligible and trial not-eligible portions of the target population (A4). These assumptions are weaker than those required when estimating treatment effects based on the treated cohort alone (where A1 is made instead of A3) or trial data alone, where we must assume that the trial and trial-eligible portions of the target population do not differ on any characteristics which predict treatment effect (A2) rather than A4.

### Simulation study

To investigate the properties of the proposed new estimator, a simulation study was conducted. The target population, P, can be split into the trial-eligible portion P_1_, and the trial not-eligible portion P_2_. We assumed that P_1_ could be further split into k mutually exclusive and exhaustive subsets P_1j_, representing those eligible for each of k trials. Using Z_i_ as an indicator for trial eligibility, Z_i_ = 1 if individual i is trial eligible, 0 otherwise, and setting P(Z_i_ = 1) = π, then the treatment effect for each individual [Δ_i_, eqn (2)] can be generated using:


(15) $$\begin{array}{l} \Delta_{i}|\;(Z_{i}=1\cap \;i\in P_{1j})\;∼\;N\left({\text{ARE}}_{1j},\;\omega^{2}\right)\\ \Delta_{i}|{\;Z}_{i}=0\;∼\;N({\text{ARE}}_{2},\nu^{2})\\ {\text{ARE}}_{1j}∼\;N({\text{ARE}}_{1},\tau^{2}) \end{array}$$

The outcomes for individuals at treatment offer γ_0, i_, can similarly be defined as follows:


(16) $$\begin{array}{l} \gamma_{0,i}=y_{i0,}{}_{R=1}=y_{i0,\;R=0}\\ \gamma_{0,i}|(Z_{i}=1\cap \;i\in P_{1j})\;∼\;N\left(\Gamma_{01,j},\;\omega_{0}{}^{2}\right)\\ \gamma_{0,i}|Z_{i}=0\;∼\;N(\Gamma_{02},\sigma_{0}{}^{2})\\ \Gamma_{01,j}∼\;N(\Gamma_{01},\tau_{0}{}^{2}) \end{array}$$

For each individual, these values are first generated and the trajectories under treatment offer are derived. The trajectory under no treatment offer is assumed to be: where t = 0 is the point where treatment would have been offered and the trajectory has two slopes. Equation (2) can be rearranged to show:


(17) $$\begin{array}{l} y_{\text{it},\;R=0}=\gamma_{0,i}+\gamma_{1}t+\varepsilon ,\;\;\;\;\;\;\;t<0\\ \gamma_{0,i}+\gamma_{2}t+\varepsilon ,\;\;\;\;\;\;\;\;t\geq 0 \end{array}$$

(18) $$y_{i\alpha }{,}_{R=1}=\Delta_{i}+{\;y}_{i\alpha ,\;R=0}$$

Assuming that the trajectory under treatment offer has a second change point at t = δ and slope γ_3_, thereafter the following expressions for the trajectory under treatment offer can be derived:


(19) $$\begin{array}{l} \text{If}\;\alpha <\delta :\;y_{\text{it},\;R=1}=y_{\text{it},\;R=0}+\frac{1}{\alpha }\Delta_{i}t,\;\;\;\;\;\;\;\;\;\;\;\;\;\;\;\;\;\;\;\;\;\;\;\;\;\;\;\;\;\;\;t<\delta \\ \;\;\;\;\;\;\;\;\;\;\;\;\;\;\;\;\;\;\;\;\;\;\;\;\;\;\;\;\;\;\;\;\;\;\;\;\;\;\;\;\;\;\;\;\;\;\;\;\;\;\;\;\;y_{\text{it},\;R=0}+\frac{1}{\alpha }\Delta_{i}\delta +\left(\gamma_{3}–\;\gamma_{2}\right)\left(t\;–\;\delta \right),\;\;t\geq \delta \\ \text{If}\;\alpha \geq \delta :\;y_{\text{it},\;R=1}=y_{\text{it},\;R=0}+\frac{1}{\delta }\Delta_{i}t+\left(\gamma_{3}–\;\gamma_{2}\right)\\ (1-\frac{\alpha }{\delta }),\;\;\;\;t<\delta \\ \;y_{\text{it},\;R=0}+\Delta_{i}+\left(\gamma_{3}–\;\gamma_{2}\right)\left(t\;–\;\alpha \right),\;\;\;\;\;\;\;\;\;\;\;t\geq \delta \end{array}$$

Expressions for the projection bias φ_α_, and generalizability bias ζ, are as follows:


(20) $$\begin{array}{l} \varphi_{\alpha }=(\gamma_{2}–\;\gamma_{1})\alpha \\ \zeta ={\;\text{ARE}}_{1}–\;\text{ARE} \end{array}$$

Trial samples for each of the k trials were generated by first assuming that the whole trial sample size, n_1_, was split evenly among the k trials so that $\frac{n1}{k}$ participants from P_1j_ were selected for each trial. For each, their outcome at treatment offer was obtained from Equation (17). Randomization, R∼Bin(1, 1/2), was generated to ensure 50:50 allocation, and their outcome at t = α was obtained using Equation (19) if R = 1 and Equation (17) if R = 0. For each participant their change score from time 0 to α was calculated, and these were aggregated to provide an estimate of effectiveness and associated standard error from each trial. A cohort sample was generated by: selecting n_2_ participants from the target population P; choosing the time points at which observations are recorded for individual i, t_sel, i_, to ensure variation in location and number of measurements; and generating potential outcomes at selected time points using Equation (19).

Input values investigated for each of the parameters are summarized in Table 1. Parameters of interest (size of datasets, projection and generalizability biases, and proportion trial eligible^27^^,^^28^) were investigated with the combinations considered determined using a Latin Squares Design^29^ in order to reduce the number of parameter combinations considered. Situations where generalizability bias, projection bias and both generalizability and projection bias simultaneously were present in the data were considered. Other parameters were set to reasonable values, based on example datasets.

**Table 1:** Input parameters used for simulation model

Parameter	Description	Values
n_1_	Combined size of all trials	1000, 3000, 10 000
k	Number of trials	4, 12, 40
n_2_	Size of cohort dataset	1500, 3000, 6000
ARE	Average effect of treatment offer in P	1
ARE_1_	Average effect of treatment offer in P_1_	0.5, 1, 1.5
	Note: values chosen to investigate ζ = -0.5, 0, 0.5	
α	Time treatment effect measured at (years)	0.25, 0.5, 1
π	Proportion of target population trial eligible	0.5, 0.7, 0.9
Γ_0_	Mean intercept in P	20
Γ_02_	Mean intercept in P_2_	20
γ_1_	Rate of decline t <0	−2
γ_2_	Rate of decline among patients not offered treatment	if α = 0.25: -4, -2, 0
	Note: values chosen to investigate φ_α_ = -0.5, 0, 0.5	if α = 0.5: -3, -2, -1
		if α = 1: -2.5, -2, -1.5
γ_3_	Rate of decline when t ≥δ in patients offered treatment	−1.9
δ	Second change point (time in years)	0.3
σ	Residual standard deviation	2
σ_0_	Intercept standard deviation	4
ν^2^	Total variation in Δ_i_ in P_1_ and in P_2_	1
τ_1_^2^	Between trial variation in P_1_	0.25

ARE is used to denote the average effect of treatment offer, since it seeks to approximate the effect of randomized treatment allocation analysed under the intention-to-treat principle.

Three competing estimators were calculated for each simulated dataset: (i) trials only estimator; (ii) cohort only estimator; and (iii) combined estimator. Estimators were compared in terms of absolute bias, standard error (SE) and mean squared error (MSE); 500 sets of data were generated for each combination of input values.

## Results

### Simulation study

**Figure 2:**
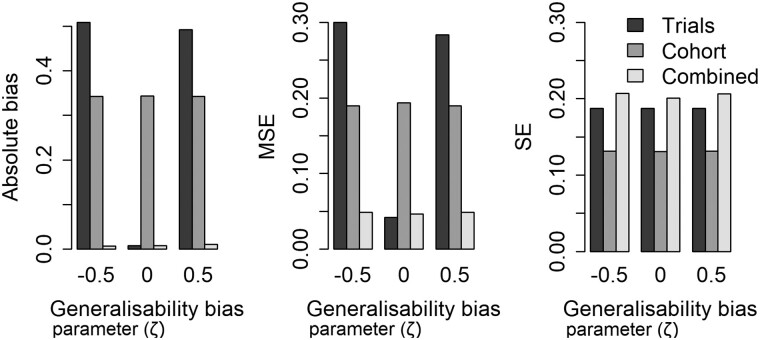
Impact of generalizability bias on performance estimators at α = 0.25

**Figure 3:**
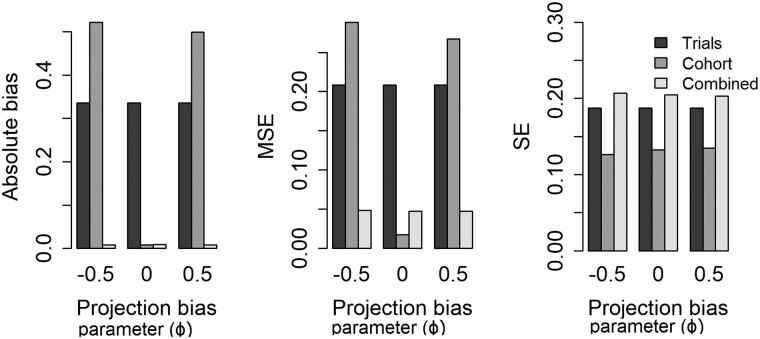
Impact of projection bias on performance of estimators at α = 0.25

The impact of varying the generalizability bias parameter (ζ) at α = 0.25 and averaged across all other parameters is summarized in Figure 2. In the presence of generalizability bias (non-zero ζ) the combined estimator is less biased and has a smaller MSE than the trials-only estimator. When there is no generalizability bias (ζ = 0), that is when the RCT estimate is representative of the whole population, the combined estimator remains unbiased but the MSE is slightly larger than the trials-only estimator. Similarly, when varying the projection bias, the combined model has lower bias and MSE than the cohort only estimator in the presence of projection bias and remains unbiased, but with slightly larger MSE when there was no projection bias (see Figure 3). Variation in the sizes of the two datasets and the trial-eligible proportion did not affect on bias estimates; however, increasing the proportion trial eligible or either of the sample sizes led to a reduction in the MSE of the combined estimator. Tables summarizing outputs from all combinations of inputs considered in the simulation study are provided in the Supplementary Material (available as Supplementary data at *IJE* online).

### Illustration using real data

Having demonstrated the favourable performance of the combined model in the simulation study, it was applied to the motivating clinical question. The recent systematic review of AChEIs in the management of dementia identified four trials estimating their effects at 12 months after treatment offer.^30–33^ Five overall eligibility criteria for these trials were established: (i) baseline age between 40 and 94 years; (ii) baseline MMSE between 10 and 26; (iii) diagnosed with Alzheimer’s or Alzheimer’s and cerebrovascular disease; (iv) participant has a reliable/responsible caregiver; and (v) participant does not have another major psychiatric disorder. Participants in the treated cohort meeting these criteria were identified. Estimates of treatment effect based on the trials only were calculated as part of the systematic review. Estimates based on the treated cohort only and using the new combined model were calculated. All three estimators suggested a modest but significant effect in favour of treatment (see Table 2). The estimate based on the combined model was lower than those based on either of the two data sources alone, demonstrating that the combined model can quantify both generalizability and projection bias even when they are both in the same direction.

**Table 2:** Comparing estimates of the average effect of the offer of cetylcholinesterase inhibitors treatment at 12 months after treatment offer in terms of the Mini Mental State Examination

	Trials only	Cohort only	Trials and cohort
ARE	1.10	1.56	0.86
SE(ARE)	0.316	0.240	0.327

ARE is used to denote the average effect of treatment offer, since it seeks to approximate the effect of randomized treatment allocation analysed under the intention-to-treat principle..

## Discussion

In this paper we have proposed and evaluated a novel estimator of treatment effectiveness which incorporates data from RCTs to improve the estimates of treatment effect available from analysis of data on a treated cohort. The performance of the novel estimator was compared with that of those based on either data source alone via a simulation study, demonstrating the model to be superior for estimating effectiveness in the presence of bias in one or both of the data sources. The model was applied to estimate the effectiveness of AChEIs 12 months after treatment offer in terms of MMSE scores, and this highlighted an important strength of the new combined model: namely that even when both of the estimates based on a single data source are biased in the same direction, the model can identify and account for these biases.

The combined model used a Bayesian framework, allowing the incorporation of data external to the current dataset in the form of informative prior distributions. This model can be considered an example of the type of bias analysis proposed by Greenland.^34^ In this combination of data sources, it is important to account for potential differences in study design and data collection features between the sources. The lack of such a mechanism is one criticism of many of the existing techniques.^35^ Others have suggested that results could be weighted to account for perceived differences in reliability of data sources,^12^^,^^13^ adjusting point estimates given anticipated bias^36^ or discounting the weight of prior information using power priors.^37^ However, each of these approaches requires substantial subjective judgement about how to make these adjustments, which can present challenges. On the other hand, the approach proposed here provides a method by which informative priors can be applied only to the proportion of the population to which they apply.

One limitation of this method is that it does not address potential differences in distribution between the trial-eligible portion of the treated cohort and the trial sample (the requirement for assumption A3 to hold). Approaches to account for these differences^38^ could be investigated in future as a possible expansion to the model proposed here, which would allow assumption A3 to be relaxed and increase the possible applications for the model. In addition, the model does not currently address the possibility that adherence rates may differ between the trial and cohort populations, instead relying on the fact that these are likely to be similar when the trials in question are pragmatic phase III trials. Future work could address this by incorporating adherence in the model; however, this would require careful definition of adherence in both data sources.

A further limitation which may be encountered in applying such a model is the need to be able to identify patient eligibility in an EMR; however, the availability of such data is increasing.^39^ Techniques such as natural language processing, constructing variables from constituent parts or the use of proxies may be required; these can increase the time and complexity of fitting this type of model. Similarly, whereas all trial reports should include details of eligibility criteria as per the CONSORT Statement,^40^ the implementation of these guidelines has been mixed and there is still need for further improvements.^41^^,^^42^ In addition, the new combined model relies on assumption A4; however, this is weaker than assumption A1 which must be made when estimating treatment effects based on only one type of data.

Assumption A4 is analogous to the one made when calculating the linearly extrapolated estimator of treatment effect in Cross Design Synthesis.^15^ The model proposed here does, however, have advantages over Cross Design Synthesis, in that it uses a treated cohort from routine data rather than a comparative observational study; such data sources are more readily and more widely available. In addition, the approach proposed here uses both data sources directly in estimating the treatment effect.

The approach has been developed for a continuous outcome measure; however, it could be expanded in future for use with in other clinical settings (e.g. where patients are expected to recover permanently as a result of treatment) or other data types (for example binary or time-to-event data). This would require careful consideration of reasonable assumptions for outcomes under control conditions in the EMR data and then using these assumptions to derive an appropriate model with a treatment effect parameter on which a prior could be placed. Simulation studies would be required to investigate the performance of such an expansion of the model. The modelling methods could also be applied in other conditions and to estimate the effectiveness of other treatments.

## Conclusion

In conclusion, in this paper we have proposed a Bayesian mixed model approach to combining data from trials and a treated cohort to estimate treatment effectiveness, and demonstrated, using a simulation study, the superiority of estimates of effectiveness produced by this model compared with those provided by either data source alone. The new model was also applied to estimate the effectiveness at 12 months after treatment offer of AChEIs in the management of dementia as measured using the MMSE. Several possible avenues for future extensions of this model have also been proposed.

## Ethics approval

The Clinical Record Interactive Search system received ethical approval from the Oxfordshire Research Ethics Committee C as an anonymized data resource.

## Supplementary Material

dyac185_Supplementary_Data

## Data Availability

The source clinical data used for this study can be made available within the secure data environment in which CRIS extracts are hosted, subject to access requirements for this environment.

## Declaration

This paper represents independent research funded by the National Institute for Health Research (NIHR) Biomedical Research Centre at South London and Maudsley NHS Foundation Trust and King’s College London as part of a PhD studentship to R.K. The views expressed are those of the authors and not necessarily those of the NHS, the NIHR or the Department of Health.
