# Supplementary material for: Borrowing strength from clinical trials in analysing longitudinal data from a treated cohort: investigating the effectiveness of acetylcholinesterase inhibitors in the management of dementia
Source: Int J Epidemiol. 2022 Oct 11;52(3):827–36. doi: 10.1093/ije/dyac185 (PMC10244047; doi:10.1093/ije/dyac185)
Supplement: dyac185_Supplementary_Data [file dyac185_supplementary_data.docx]

Supplementary material

1. **Example R code and BUGs models**

*R code to fit the three alternatives*

**# 1. Loading relevant R commands ####**

library(metafor) # use for meta-analysis

library(BRugs) # use for Bayesian models

**# 2. Fitting model to Trials data only ####**

meta_trials <- rma.uni(yi=D_rct, sei=SE_rct, data=rct_data)

# Note: the rma.uni command fits a meta-analysis to the RCT data assuming that results from individual trials have been organised into a data frame called rct_data with effect estimates in a column called D_rct and standard errors in a column called SE_rct

ARE_trial <- as.numeric(meta_trials[1]) # trials only ARE estimator

SE_trial <- as.numeric(meta_trials[2]) # SE of trials only ARE estimator

**# 3. Fitting model to cohort data only ####**

**# 3.1 Identifying cohort data ####**

id <- obs_data[,1] # patient identifier, one per patient

vt <- obs_data[,2] # times of outcome measurement (measured from treatment offer)

mmse <- obs_data[,3] # outcomes measured

POINTS <- nrow(obs_data) # number of data points in model

PEOPLE <- length(unique(id)) # number of patients in model

**# 3.2 Prior distributions ####**

mu.BF<-c(0,0,0,0) # mean of prior distribution

tau.BF<-matrix(c( 0.0001,0,0,0,

0,0.0001,0,0,

0,0,0.0001,0,

0,0,0,0.0001),nrow=4) # covariance of prior

**# 3.3 Model input and output ####**

data_obs<-list("POINTS","PEOPLE","mmse","vt","id","alpha","tau.BF","mu.BF")

# Combines all data needed to fit the model into a single list

params_obs<-list("theta")

# parameters to save outputs from the model for, can add others to this list

inits_obs<-list(list(BF=c(22,-1,2,-0.9),

delta=0.45,sigma=3,sigma.b0=5,sigma.b2=2.1,rho=0.5,

B=matrix(rnorm(2*PEOPLE,0,2),nrow=PEOPLE,ncol=2)),

list(BF=c(18,-3,0,-2.9),

delta=0.15,sigma=1,sigma.b0=3,sigma.b2=0.1,rho=-0.5,

B=matrix(rnorm(2*PEOPLE,0,2),nrow=PEOPLE,ncol=2)))

# initial values for model – some plausible values are needed as a starting point to fit the model

**# 3.4 Fitting model and examining output ####**

model_obs<-BRugsFit("obs_model.txt",data=data_obs,inits=inits_obs,

numChains=2,parametersToSave=params_obs,

nBurnin=burn_in,nIter=iterations,nThin=thin)

# Bayesian model fitting – this command calls to BUGS (and therefore requires this is installed on your machine. The model this refers to is provided later in this supplementary material

ARE_obs<-model_obs[[1]][1,1] # cohort only ARE estimator

SE_obs<-model_obs[[1]][1,2] # SE of cohort only ARE estimator

**# 4. Fitting model to both data sources ####**

**# 4.1 Informative prior and trial eligibility indicator ####**

mu_theta<-ARE_trial # informative prior mean – based on trials only data

tau_theta<-1/(SE_trial^2) # informative prior precision – based on trials only data

mu_bf<-c(0,0,mu_theta,0,0,0,0,0,0) # mean of informative prior dstn

tau_bf<-matrix(c( 0.0001,0,0,0,0,0,0,0,0,

0,0.0001,0,0,0,0,0,0,0,

0,0,tau_theta,0,0,0,0,0,0,

0,0,0,0.0001,0,0,0,0,0,

0,0,0,0,0.0001,0,0,0,0,

0,0,0,0,0,0.0001,0,0,0,

0,0,0,0,0,0,0.0001,0,0,

0,0,0,0,0,0,0,0.0001,0,

0,0,0,0,0,0,0,0,0.0001), nrow=9) # covariance of informative prior distribution

S <- obs_data[,4] # where this is a variable that indicates for each row of the cohort data whether or not the patient this row refers to met trial eligibility criteria

**# 4.2 Model input and output ####**

data_comb<-list("POINTS", "PEOPLE", "mmse", "vt", "id", "alpha", "mu_bf", "tau_bf", "S")

# combines all the data needed to fit the model into a single list

params_comb<-list("ARE","ARE1","ARE2")

# parameters to save from model, can add others to this list

inits_comb<-list(list(BF=c(18,-3,-0.5,-2.9,22,-1,2.5,-2,-1),

delta=0.15,pi=0.55,sigma=1,sigma.b0=3,sigma.b2=0.1,rho=-0.5,

B=matrix(rnorm(2*PEOPLE,0,2),nrow=PEOPLE,ncol=2)),

list(BF=c(22,-1,1.5,-0.9,18,-3,0.5,-2.9,1),

delta=0.45,pi=0.95,sigma=3,sigma.b0=5,sigma.b2=2.1,rho=0.5,

B=matrix(rnorm(2*PEOPLE,0,2),nrow=PEOPLE,ncol=2)))

# initial values for model – some plausible values are needed as a starting point to fit the model

**# 4.3 Model fitting and outputs ####**

model_comb<-BRugsFit("comb_model.txt",data=data_comb,inits=inits_comb,

numChains=2,parametersToSave=params_comb,

nBurnin=burn_in,nIter=iterations,nThin=thin)

# Bayesian model fitting – this command calls to BUGS (and therefore requires this is installed on your machine. The model this refers to is provided later in this supplementary material

ARE_comb<-model_comb[[1]][1,1] # combined estimator of ARE

SE_comb<-model_comb[[1]][1,2] # SE of combined ARE estimator

ARE1_comb<-model_comb[[1]][2,1] # estimator of ARE in P1

SE1_comb<-model_comb[[1]][2,2] # SE of ARE in P1 estimator

ARE2_comb<-model_comb[[1]][3,1] # estimator of ARE in P2

SE2_comb<-model_comb[[1]][3,2] # SE of ARE in P2 estimator

*BUGS models*

Cohort only model, used as obs_model.txt in the R code provided above:

model{

for(i in 1:POINTS){

mmse[i] ~ dnorm(mu[i], tau)

mu[i] <- beta0 + b0[id[i]] + beta1*(t1[i] + c1*t2[i]) +

(theta + b2[id[i]])*c2*t2[i] + beta3*(t3[i] + c3*t2[i])

t1[i] <- vt[i]*step(0-vt[i])

t2[i] <- vt[i]*step(vt[i]-0)*step(delta-vt[i]) +

delta*step(vt[i]-delta)

t3[i] <- (vt[i]-delta)*step(vt[i]-delta)

}

c1 <- 1*step(delta-alpha) + (alpha/delta)*step(alpha-delta)

c2 <- (1/alpha)*step(delta-alpha) + (1/delta)*step(alpha-delta)

c3 <- (1 -(alpha/delta))*step(alpha-delta)

beta0 <- BF[1]

beta1 <- BF[2]

theta <- BF[3]

beta3 <- BF[4]

BF[1:4] ~ dmnorm(mu.BF[1:4], tau.BF[,])

tau <- 1/pow(sigma,2)

sigma ~ dunif(0,100)

delta ~ dunif(0,3)

for(j in 1:PEOPLE){

b0[j] <- B[j,1]

b2[j] <- B[j,2]

B[j,1:2] ~ dmnorm(B.hat[j,], Tau.B[,])

B.hat[j,1] <- 0

B.hat[j,2] <- 0

}

Tau.B[1:2,1:2] <- inverse(Sigma.B[,])

Sigma.B[1,1] <- pow(sigma.b0,2)

sigma.b0 ~ dunif(0, 100)

Sigma.B[2,2] <- pow(sigma.b2,2)

sigma.b2 ~ dunif(0, 100)

Sigma.B[1,2] <- rho*sigma.b0*sigma.b2

Sigma.B[2,1] <- rho*sigma.b0*sigma.b2

rho ~ dunif(-1,1)

}

Combined model, referred to as comb_model.txt in the above:

model{

for(i in 1:POINTS){

mmse[i] ~ dnorm(mu[i], tau)

mu[i] <- (beta01*step(S[id[i]]-0.5)+beta02*step(0.5-S[id[i]]))+

b0[id[i]] + (beta11*step(S[id[i]]-0.5)+

beta12*step(0.5-S[id[i]]))*(t1[i] + c1*t2[i]) +

(theta1*step(S[id[i]]-0.5) +

theta2*step(0.5-S[id[i]])+b2[id[i]])*c2*t2[i] +

(beta31*step(S[id[i]]-0.5) +

beta32*step(0.5-S[id[i]]))*(t3[i]+c3*t2[i])

t1[i] <- vt[i]*step(0-vt[i])

t2[i] <- vt[i]*step(vt[i]-0)*step(delta-vt[i]) +

delta*step(vt[i]-delta)

t3[i] <- (vt[i]-delta)*step(vt[i]-delta)

}

c1 <- 1*step(delta-alpha) + (alpha/delta)*step(alpha-delta)

c2 <- (1/alpha)*step(delta-alpha) + (1/delta)*step(alpha-delta)

c3 <- (1 -(alpha/delta))*step(alpha-delta)

beta01 <- BF[1]

beta11 <- BF[2]

theta1 <- ARE1 + f

ARE1 <- BF[3]

beta31 <- BF[4]

beta02 <- BF[5]

beta12 <- BF[6]

theta2 <- ARE2 + f

ARE2 <- BF[7]

beta32 <- BF[8]

f <- BF[9]

BF[1:9] ~ dmnorm(mu_bf[1:9],tau_bf[,])

delta ~ dunif(0,3)

tau <- 1/pow(sigma,2)

sigma ~ dunif(0,100)

for(j in 1:PEOPLE){

b0[j] <- B[j,1]

b2[j] <- B[j,2]

B[j,1:2] ~ dmnorm(B.hat[j,], Tau.B[,])

B.hat[j,1] <- 0

B.hat[j,2] <- 0

S[j] ~ dbin(pi,1)

}

Tau.B[1:2,1:2] <- inverse(Sigma.B[,])

Sigma.B[1,1] <- pow(sigma.b0,2)

sigma.b0 ~ dunif(0, 100)

Sigma.B[2,2] <- pow(sigma.b2,2)

sigma.b2 ~ dunif(0, 100)

Sigma.B[1,2] <- rho*sigma.b0*sigma.b2

Sigma.B[2,1] <- rho*sigma.b0*sigma.b2

rho ~ dunif(-1,1)

pi ~ dunif(0,1)

ARE<- pi*ARE1 + (1-pi)*ARE2

gen <- ARE1-ARE

}

1. **Simulations study results**

Results from all input combination explored in the simulation study are provided. Simulations investigated the ability of the model to estimate treatment effects at 3, 6, and 12 months after treatment offer. Cohort sample and trial sample sizes were both explored at 6 months. Due to computing time cohort sample size was fixed at 3 months (n_2_=3000) and trial sample size was fixed at 12 months (n_1_=1000).

**Table S1**: Simulation study results estimating treatment effects at 3 months after treatment offer (n_2_=3000)

| **ζ** | **φ** | **π** | **n_1_** | **Absolute bias** | | | **MSE** | | | **SE** | | | **Projection interval** | **Generalisabilty interval** |
| --- | --- | --- | --- | --- | --- | --- | --- | --- | --- | --- | --- | --- | --- | --- |
|  |  |  |  | **Trials** | **Cohort** | **Combined** | **Trials** | **Cohort** | **Combined** | **Trials** | **Cohort** | **Combined** |  |  |
| -0.5 | -0.5 | 50 | 3000 | 0.508 | 0.536 | 0.019 | 0.289 | 0.298 | 0.040 | 0.177 | 0.103 | 0.200 | 0.950 | 0.960 |
| -0.5 | -0.5 | 70 | 10000 | 0.506 | 0.522 | 0.004 | 0.266 | 0.282 | 0.017 | 0.099 | 0.099 | 0.129 | 0.954 | 0.970 |
| -0.5 | -0.5 | 90 | 1000 | 0.512 | 0.504 | 0.003 | 0.347 | 0.265 | 0.087 | 0.293 | 0.103 | 0.295 | 0.920 | 0.954 |
| -0.5 | 0 | 50 | 1000 | 0.512 | 0.010 | 0.016 | 0.347 | 0.013 | 0.095 | 0.293 | 0.115 | 0.308 | 0.928 | 0.938 |
| -0.5 | 0 | 70 | 3000 | 0.508 | 0.024 | 0.001 | 0.289 | 0.013 | 0.039 | 0.177 | 0.113 | 0.197 | 0.938 | 0.958 |
| -0.5 | 0 | 90 | 10000 | 0.506 | 0.001 | 0.002 | 0.266 | 0.013 | 0.012 | 0.099 | 0.112 | 0.109 | 0.962 | 0.950 |
| -0.5 | 0.5 | 50 | 10000 | 0.506 | 0.496 | 0.009 | 0.266 | 0.261 | 0.017 | 0.099 | 0.123 | 0.131 | 0.962 | 0.936 |
| -0.5 | 0.5 | 70 | 1000 | 0.512 | 0.487 | 0.004 | 0.347 | 0.251 | 0.091 | 0.293 | 0.120 | 0.302 | 0.924 | 0.944 |
| -0.5 | 0.5 | 90 | 3000 | 0.508 | 0.502 | 0.000 | 0.289 | 0.267 | 0.033 | 0.177 | 0.120 | 0.183 | 0.934 | 0.958 |
| 0 | -0.5 | 50 | 10000 | 0.006 | 0.523 | 0.011 | 0.010 | 0.285 | 0.016 | 0.099 | 0.107 | 0.126 | 0.970 | 0.954 |
| 0 | -0.5 | 70 | 1000 | 0.012 | 0.523 | 0.007 | 0.086 | 0.285 | 0.090 | 0.293 | 0.108 | 0.300 | 0.924 | 0.942 |
| 0 | -0.5 | 90 | 3000 | 0.008 | 0.525 | 0.007 | 0.031 | 0.287 | 0.033 | 0.177 | 0.108 | 0.181 | 0.948 | 0.950 |
| 0 | 0 | 50 | 3000 | 0.008 | 0.001 | 0.013 | 0.031 | 0.013 | 0.038 | 0.177 | 0.115 | 0.195 | 0.960 | 0.956 |
| 0 | 0 | 70 | 10000 | 0.006 | 0.002 | 0.001 | 0.010 | 0.013 | 0.015 | 0.099 | 0.114 | 0.121 | 0.956 | 0.936 |
| 0 | 0 | 90 | 1000 | 0.012 | 0.002 | 0.011 | 0.086 | 0.014 | 0.086 | 0.293 | 0.117 | 0.293 | 0.932 | 0.944 |
| 0 | 0.5 | 50 | 1000 | 0.012 | 0.504 | 0.017 | 0.086 | 0.270 | 0.094 | 0.293 | 0.124 | 0.306 | 0.934 | 0.950 |
| 0 | 0.5 | 70 | 3000 | 0.008 | 0.508 | 0.002 | 0.031 | 0.273 | 0.038 | 0.177 | 0.120 | 0.194 | 0.954 | 0.938 |
| 0 | 0.5 | 90 | 10000 | 0.006 | 0.505 | 0.005 | 0.010 | 0.270 | 0.011 | 0.099 | 0.125 | 0.104 | 0.944 | 0.950 |
| 0.5 | -0.5 | 50 | 1000 | 0.488 | 0.535 | 0.014 | 0.324 | 0.297 | 0.094 | 0.293 | 0.105 | 0.307 | 0.938 | 0.974 |
| 0.5 | -0.5 | 70 | 3000 | 0.492 | 0.524 | 0.001 | 0.274 | 0.285 | 0.039 | 0.177 | 0.099 | 0.197 | 0.952 | 0.968 |
| 0.5 | -0.5 | 90 | 10000 | 0.494 | 0.504 | 0.012 | 0.254 | 0.264 | 0.013 | 0.099 | 0.101 | 0.113 | 0.942 | 0.962 |
| 0.5 | 0 | 50 | 10000 | 0.494 | 0.010 | 0.015 | 0.254 | 0.014 | 0.018 | 0.099 | 0.119 | 0.133 | 0.974 | 0.960 |
| 0.5 | 0 | 70 | 1000 | 0.488 | 0.023 | 0.006 | 0.324 | 0.014 | 0.091 | 0.293 | 0.115 | 0.301 | 0.924 | 0.954 |
| 0.5 | 0 | 90 | 3000 | 0.492 | 0.000 | 0.013 | 0.274 | 0.012 | 0.034 | 0.177 | 0.110 | 0.185 | 0.938 | 0.952 |
| 0.5 | 0.5 | 50 | 3000 | 0.492 | 0.498 | 0.016 | 0.274 | 0.264 | 0.039 | 0.177 | 0.124 | 0.198 | 0.960 | 0.952 |
| 0.5 | 0.5 | 70 | 10000 | 0.494 | 0.485 | 0.003 | 0.254 | 0.250 | 0.016 | 0.099 | 0.123 | 0.125 | 0.960 | 0.948 |
| 0.5 | 0.5 | 90 | 1000 | 0.488 | 0.502 | 0.017 | 0.324 | 0.266 | 0.088 | 0.293 | 0.119 | 0.296 | 0.928 | 0.952 |

**Table S2:** Simulation study results estimating treatment effects at 6 months after treatment offer

| **ζ** | **φ** | **π** | **n_2_** | **n_1_** | **Absolute bias** | | | **MSE** | | | **SE** | | | **Projection interval** | **Gen. interval** |
| --- | --- | --- | --- | --- | --- | --- | --- | --- | --- | --- | --- | --- | --- | --- | --- |
|  |  |  |  |  | **Trials** | **Cohort** | **Combined** | **Trials** | **Cohort** | **Combined** | **Trials** | **Cohort** | **Combined** |  |  |
| -0.5 | -0.5 | 50 | 1000 | 1500 | 0.507 | 0.528 | 0.014 | 0.361 | 0.326 | 0.139 | 0.323 | 0.219 | 0.373 | 0.920 | 0.930 |
| -0.5 | -0.5 | 50 | 3000 | 3000 | 0.508 | 0.521 | 0.016 | 0.289 | 0.291 | 0.048 | 0.177 | 0.139 | 0.218 | 0.954 | 0.940 |
| -0.5 | -0.5 | 50 | 10000 | 6000 | 0.497 | 0.505 | 0.001 | 0.257 | 0.265 | 0.017 | 0.097 | 0.099 | 0.131 | 0.964 | 0.960 |
| -0.5 | -0.5 | 70 | 1000 | 6000 | 0.492 | 0.511 | 0.006 | 0.333 | 0.271 | 0.098 | 0.302 | 0.102 | 0.312 | 0.890 | 0.948 |
| -0.5 | -0.5 | 70 | 3000 | 1500 | 0.511 | 0.509 | 0.012 | 0.293 | 0.304 | 0.052 | 0.179 | 0.214 | 0.228 | 0.938 | 0.934 |
| -0.5 | -0.5 | 70 | 10000 | 3000 | 0.506 | 0.518 | 0.002 | 0.266 | 0.287 | 0.020 | 0.099 | 0.138 | 0.141 | 0.958 | 0.922 |
| -0.5 | -0.5 | 90 | 1000 | 3000 | 0.512 | 0.507 | 0.007 | 0.347 | 0.276 | 0.088 | 0.293 | 0.138 | 0.296 | 0.928 | 0.960 |
| -0.5 | -0.5 | 90 | 3000 | 6000 | 0.507 | 0.495 | 0.003 | 0.291 | 0.255 | 0.036 | 0.184 | 0.099 | 0.189 | 0.918 | 0.934 |
| -0.5 | -0.5 | 90 | 10000 | 1500 | 0.513 | 0.483 | 0.008 | 0.273 | 0.279 | 0.015 | 0.097 | 0.212 | 0.124 | 0.956 | 0.950 |
| -0.5 | 0 | 50 | 1000 | 6000 | 0.492 | 0.002 | 0.007 | 0.333 | 0.009 | 0.098 | 0.302 | 0.095 | 0.312 | 0.920 | 0.962 |
| -0.5 | 0 | 50 | 3000 | 1500 | 0.511 | 0.010 | 0.010 | 0.293 | 0.038 | 0.069 | 0.179 | 0.195 | 0.262 | 0.952 | 0.930 |
| -0.5 | 0 | 50 | 10000 | 3000 | 0.506 | 0.006 | 0.014 | 0.266 | 0.016 | 0.025 | 0.099 | 0.126 | 0.158 | 0.960 | 0.944 |
| -0.5 | 0 | 70 | 1000 | 3000 | 0.512 | 0.001 | 0.003 | 0.347 | 0.017 | 0.093 | 0.293 | 0.130 | 0.305 | 0.922 | 0.928 |
| -0.5 | 0 | 70 | 3000 | 6000 | 0.507 | 0.002 | 0.008 | 0.291 | 0.009 | 0.040 | 0.184 | 0.094 | 0.199 | 0.910 | 0.946 |
| -0.5 | 0 | 70 | 10000 | 1500 | 0.513 | 0.019 | 0.013 | 0.273 | 0.041 | 0.027 | 0.097 | 0.201 | 0.162 | 0.936 | 0.928 |
| -0.5 | 0 | 90 | 1000 | 1500 | 0.507 | 0.017 | 0.003 | 0.361 | 0.045 | 0.110 | 0.323 | 0.212 | 0.331 | 0.908 | 0.956 |
| -0.5 | 0 | 90 | 3000 | 3000 | 0.508 | 0.007 | 0.003 | 0.289 | 0.019 | 0.034 | 0.177 | 0.138 | 0.184 | 0.946 | 0.958 |
| -0.5 | 0 | 90 | 10000 | 6000 | 0.497 | 0.005 | 0.006 | 0.257 | 0.010 | 0.011 | 0.097 | 0.099 | 0.105 | 0.950 | 0.928 |
| -0.5 | 0.5 | 50 | 1000 | 3000 | 0.512 | 0.493 | 0.019 | 0.347 | 0.259 | 0.103 | 0.293 | 0.126 | 0.320 | 0.938 | 0.942 |
| -0.5 | 0.5 | 50 | 3000 | 6000 | 0.507 | 0.498 | 0.009 | 0.291 | 0.257 | 0.040 | 0.184 | 0.096 | 0.20 | 0.936 | 0.962 |
| -0.5 | 0.5 | 50 | 10000 | 1500 | 0.513 | 0.513 | 0.011 | 0.273 | 0.301 | 0.053 | 0.097 | 0.194 | 0.229 | 0.938 | 0.932 |
| -0.5 | 0.5 | 70 | 1000 | 1500 | 0.507 | 0.521 | 0.006 | 0.361 | 0.311 | 0.123 | 0.323 | 0.200 | 0.351 | 0.908 | 0.936 |
| -0.5 | 0.5 | 70 | 3000 | 3000 | 0.508 | 0.501 | 0.001 | 0.289 | 0.267 | 0.042 | 0.177 | 0.130 | 0.206 | 0.952 | 0.928 |
| -0.5 | 0.5 | 70 | 10000 | 6000 | 0.497 | 0.502 | 0.001 | 0.257 | 0.261 | 0.014 | 0.097 | 0.094 | 0.118 | 0.962 | 0.944 |
| -0.5 | 0.5 | 90 | 1000 | 6000 | 0.492 | 0.505 | 0.011 | 0.333 | 0.265 | 0.093 | 0.302 | 0.100 | 0.305 | 0.900 | 0.924 |
| -0.5 | 0.5 | 90 | 3000 | 1500 | 0.511 | 0.518 | 0.006 | 0.293 | 0.313 | 0.040 | 0.179 | 0.211 | 0.199 | 0.950 | 0.952 |
| -0.5 | 0.5 | 90 | 10000 | 3000 | 0.506 | 0.494 | 0.002 | 0.266 | 0.263 | 0.012 | 0.099 | 0.137 | 0.111 | 0.964 | 0.958 |
| 0 | -0.5 | 50 | 1000 | 6000 | 0.008 | 0.503 | 0.007 | 0.091 | 0.262 | 0.097 | 0.302 | 0.096 | 0.312 | 0.926 | 0.956 |
| 0 | -0.5 | 50 | 3000 | 1500 | 0.011 | 0.527 | 0.011 | 0.032 | 0.324 | 0.064 | 0.179 | 0.217 | 0.254 | 0.940 | 0.940 |
| 0 | -0.5 | 50 | 10000 | 3000 | 0.006 | 0.515 | 0.013 | 0.010 | 0.283 | 0.025 | 0.099 | 0.133 | 0.157 | 0.954 | 0.936 |
| 0 | -0.5 | 70 | 1000 | 3000 | 0.012 | 0.510 | 0.005 | 0.086 | 0.279 | 0.093 | 0.293 | 0.136 | 0.305 | 0.926 | 0.932 |
| 0 | -0.5 | 70 | 3000 | 6000 | 0.007 | 0.5 | 0.008 | 0.034 | 0.259 | 0.040 | 0.184 | 0.092 | 0.199 | 0.916 | 0.948 |
| 0 | -0.5 | 70 | 10000 | 1500 | 0.013 | 0.516 | 0.014 | 0.010 | 0.315 | 0.025 | 0.097 | 0.221 | 0.156 | 0.930 | 0.936 |
| 0 | -0.5 | 90 | 1000 | 1500 | 0.007 | 0.520 | 0.005 | 0.104 | 0.317 | 0.108 | 0.323 | 0.218 | 0.329 | 0.920 | 0.958 |
| 0 | -0.5 | 90 | 3000 | 3000 | 0.008 | 0.516 | 0.006 | 0.031 | 0.284 | 0.034 | 0.177 | 0.133 | 0.183 | 0.940 | 0.948 |
| 0 | -0.5 | 90 | 10000 | 6000 | 0.003 | 0.500 | 0.005 | 0.009 | 0.259 | 0.011 | 0.097 | 0.092 | 0.102 | 0.952 | 0.946 |
| 0 | 0 | 50 | 1000 | 3000 | 0.012 | 0.005 | 0.019 | 0.086 | 0.016 | 0.102 | 0.293 | 0.125 | 0.320 | 0.940 | 0.944 |
| 0 | 0 | 50 | 3000 | 6000 | 0.007 | 0.001 | 0.008 | 0.034 | 0.009 | 0.040 | 0.184 | 0.095 | 0.201 | 0.936 | 0.958 |
| 0 | 0 | 50 | 10000 | 1500 | 0.013 | 0.011 | 0.012 | 0.010 | 0.037 | 0.052 | 0.097 | 0.192 | 0.227 | 0.940 | 0.936 |
| 0 | 0 | 70 | 1000 | 1500 | 0.007 | 0.020 | 0.007 | 0.104 | 0.040 | 0.123 | 0.323 | 0.198 | 0.351 | 0.910 | 0.928 |
| 0 | 0 | 70 | 3000 | 3000 | 0.008 | 0.001 | 0.000 | 0.031 | 0.016 | 0.042 | 0.177 | 0.127 | 0.205 | 0.948 | 0.930 |
| 0 | 0 | 70 | 10000 | 6000 | 0.003 | 0.001 | 0.001 | 0.009 | 0.008 | 0.014 | 0.097 | 0.090 | 0.118 | 0.962 | 0.942 |
| 0 | 0 | 90 | 1000 | 6000 | 0.008 | 0.001 | 0.010 | 0.091 | 0.008 | 0.093 | 0.302 | 0.091 | 0.304 | 0.898 | 0.940 |
| 0 | 0 | 90 | 3000 | 1500 | 0.011 | 0.017 | 0.008 | 0.032 | 0.038 | 0.038 | 0.179 | 0.194 | 0.194 | 0.948 | 0.956 |
| 0 | 0 | 90 | 10000 | 3000 | 0.006 | 0.005 | 0.004 | 0.010 | 0.016 | 0.012 | 0.099 | 0.125 | 0.109 | 0.958 | 0.946 |
| 0 | 0.5 | 50 | 1000 | 1500 | 0.007 | 0.513 | 0.005 | 0.104 | 0.300 | 0.135 | 0.323 | 0.191 | 0.367 | 0.930 | 0.932 |
| 0 | 0.5 | 50 | 3000 | 3000 | 0.008 | 0.495 | 0.015 | 0.031 | 0.260 | 0.048 | 0.177 | 0.125 | 0.218 | 0.960 | 0.938 |
| 0 | 0.5 | 50 | 10000 | 6000 | 0.003 | 0.498 | 0.001 | 0.009 | 0.257 | 0.017 | 0.097 | 0.095 | 0.130 | 0.966 | 0.956 |
| 0 | 0.5 | 70 | 1000 | 6000 | 0.008 | 0.501 | 0.006 | 0.091 | 0.259 | 0.097 | 0.302 | 0.090 | 0.312 | 0.904 | 0.940 |
| 0 | 0.5 | 70 | 3000 | 1500 | 0.011 | 0.522 | 0.011 | 0.032 | 0.311 | 0.051 | 0.179 | 0.197 | 0.225 | 0.938 | 0.924 |
| 0 | 0.5 | 70 | 10000 | 3000 | 0.006 | 0.502 | 0.001 | 0.010 | 0.267 | 0.020 | 0.099 | 0.126 | 0.140 | 0.956 | 0.924 |
| 0 | 0.5 | 90 | 1000 | 3000 | 0.012 | 0.495 | 0.010 | 0.086 | 0.261 | 0.087 | 0.293 | 0.125 | 0.295 | 0.932 | 0.944 |
| 0 | 0.5 | 90 | 3000 | 6000 | 0.007 | 0.501 | 0.005 | 0.034 | 0.259 | 0.036 | 0.184 | 0.091 | 0.189 | 0.922 | 0.944 |
| 0 | 0.5 | 90 | 10000 | 1500 | 0.013 | 0.519 | 0.011 | 0.010 | 0.306 | 0.014 | 0.097 | 0.192 | 0.116 | 0.948 | 0.956 |
| 0.5 | -0.5 | 50 | 1000 | 3000 | 0.488 | 0.519 | 0.019 | 0.324 | 0.289 | 0.102 | 0.293 | 0.140 | 0.320 | 0.940 | 0.938 |
| 0.5 | -0.5 | 50 | 3000 | 6000 | 0.493 | 0.504 | 0.009 | 0.277 | 0.264 | 0.040 | 0.184 | 0.100 | 0.200 | 0.932 | 0.954 |
| 0.5 | -0.5 | 50 | 10000 | 1500 | 0.487 | 0.527 | 0.002 | 0.246 | 0.324 | 0.054 | 0.097 | 0.216 | 0.232 | 0.936 | 0.934 |
| 0.5 | -0.5 | 70 | 1000 | 1500 | 0.493 | 0.504 | 0.006 | 0.347 | 0.301 | 0.123 | 0.323 | 0.217 | 0.351 | 0.912 | 0.936 |
| 0.5 | -0.5 | 70 | 3000 | 3000 | 0.492 | 0.519 | 0.001 | 0.274 | 0.289 | 0.042 | 0.177 | 0.139 | 0.205 | 0.950 | 0.928 |
| 0.5 | -0.5 | 70 | 10000 | 6000 | 0.503 | 0.512 | 0.002 | 0.262 | 0.272 | 0.014 | 0.097 | 0.101 | 0.118 | 0.964 | 0.944 |
| 0.5 | -0.5 | 90 | 1000 | 6000 | 0.508 | 0.503 | 0.009 | 0.349 | 0.263 | 0.093 | 0.302 | 0.101 | 0.304 | 0.896 | 0.956 |
| 0.5 | -0.5 | 90 | 3000 | 1500 | 0.489 | 0.481 | 0.011 | 0.272 | 0.275 | 0.039 | 0.179 | 0.209 | 0.197 | 0.944 | 0.952 |
| 0.5 | -0.5 | 90 | 10000 | 3000 | 0.494 | 0.507 | 0.007 | 0.254 | 0.275 | 0.013 | 0.099 | 0.134 | 0.114 | 0.956 | 0.944 |
| 0.5 | 0 | 50 | 1000 | 1500 | 0.493 | 0.011 | 0.004 | 0.347 | 0.038 | 0.135 | 0.323 | 0.193 | 0.367 | 0.928 | 0.938 |
| 0.5 | 0 | 50 | 3000 | 3000 | 0.492 | 0.004 | 0.015 | 0.274 | 0.016 | 0.048 | 0.177 | 0.127 | 0.218 | 0.954 | 0.938 |
| 0.5 | 0 | 50 | 10000 | 6000 | 0.503 | 0.001 | 0.001 | 0.262 | 0.009 | 0.017 | 0.097 | 0.097 | 0.131 | 0.964 | 0.956 |
| 0.5 | 0 | 70 | 1000 | 6000 | 0.508 | 0.000 | 0.007 | 0.349 | 0.009 | 0.098 | 0.302 | 0.092 | 0.313 | 0.896 | 0.940 |
| 0.5 | 0 | 70 | 3000 | 1500 | 0.489 | 0.021 | 0.012 | 0.272 | 0.041 | 0.051 | 0.179 | 0.203 | 0.225 | 0.942 | 0.938 |
| 0.5 | 0 | 70 | 10000 | 3000 | 0.494 | 0.001 | 0.000 | 0.254 | 0.017 | 0.020 | 0.099 | 0.130 | 0.140 | 0.954 | 0.926 |
| 0.5 | 0 | 90 | 1000 | 3000 | 0.488 | 0.006 | 0.012 | 0.324 | 0.018 | 0.088 | 0.293 | 0.134 | 0.297 | 0.926 | 0.944 |
| 0.5 | 0 | 90 | 3000 | 6000 | 0.493 | 0.003 | 0.006 | 0.277 | 0.010 | 0.036 | 0.184 | 0.101 | 0.190 | 0.918 | 0.956 |
| 0.5 | 0 | 90 | 10000 | 1500 | 0.487 | 0.021 | 0.013 | 0.246 | 0.044 | 0.015 | 0.097 | 0.208 | 0.123 | 0.960 | 0.950 |
| 0.5 | 0.5 | 50 | 1000 | 6000 | 0.508 | 0.499 | 0.006 | 0.349 | 0.258 | 0.097 | 0.302 | 0.097 | 0.312 | 0.920 | 0.956 |
| 0.5 | 0.5 | 50 | 3000 | 1500 | 0.489 | 0.513 | 0.008 | 0.272 | 0.300 | 0.068 | 0.179 | 0.192 | 0.261 | 0.948 | 0.940 |
| 0.5 | 0.5 | 50 | 10000 | 3000 | 0.494 | 0.496 | 0.014 | 0.254 | 0.262 | 0.025 | 0.099 | 0.127 | 0.158 | 0.962 | 0.946 |
| 0.5 | 0.5 | 70 | 1000 | 3000 | 0.488 | 0.499 | 0.005 | 0.324 | 0.266 | 0.094 | 0.293 | 0.130 | 0.307 | 0.918 | 0.930 |
| 0.5 | 0.5 | 70 | 3000 | 6000 | 0.493 | 0.500 | 0.008 | 0.277 | 0.259 | 0.040 | 0.184 | 0.093 | 0.199 | 0.914 | 0.936 |
| 0.5 | 0.5 | 70 | 10000 | 1500 | 0.487 | 0.523 | 0.014 | 0.246 | 0.314 | 0.027 | 0.097 | 0.202 | 0.164 | 0.942 | 0.934 |
| 0.5 | 0.5 | 90 | 1000 | 1500 | 0.493 | 0.521 | 0.007 | 0.347 | 0.315 | 0.109 | 0.323 | 0.208 | 0.330 | 0.918 | 0.944 |
| 0.5 | 0.5 | 90 | 3000 | 3000 | 0.492 | 0.494 | 0.008 | 0.274 | 0.262 | 0.035 | 0.177 | 0.134 | 0.186 | 0.954 | 0.944 |
| 0.5 | 0.5 | 90 | 10000 | 6000 | 0.503 | 0.497 | 0.003 | 0.262 | 0.258 | 0.011 | 0.097 | 0.101 | 0.104 | 0.950 | 0.954 |

**Table S3:** Simulation study results estimating treatment effects at 12 months after treatment offer (n_1_=1000)

| **ζ** | **φ** | **π** | **n_2_** | **Absolute bias** | | | **MSE** | | | **SE** | | | **Projection interval** | **Generalisability interval** |
| --- | --- | --- | --- | --- | --- | --- | --- | --- | --- | --- | --- | --- | --- | --- |
|  |  |  |  | **Trials** | **Cohort** | **Combined** | **Trials** | **Cohort** | **Combined** | **Trials** | **Cohort** | **Combined** |  |  |
| -0.5 | -0.5 | 50 | 1500 | 0.507 | 0.545 | 0.037 | 0.361 | 0.408 | 0.207 | 0.323 | 0.332 | 0.454 | 0.944 | 0.930 |
| -0.5 | -0.5 | 70 | 6000 | 0.492 | 0.502 | 0.004 | 0.333 | 0.273 | 0.105 | 0.302 | 0.146 | 0.325 | 0.92 | 0.942 |
| -0.5 | -0.5 | 90 | 3000 | 0.512 | 0.506 | 0.005 | 0.347 | 0.300 | 0.090 | 0.293 | 0.210 | 0.299 | 0.932 | 0.950 |
| -0.5 | 0.5 | 50 | 3000 | 0.512 | 0.494 | 0.022 | 0.347 | 0.283 | 0.128 | 0.293 | 0.199 | 0.356 | 0.944 | 0.942 |
| -0.5 | 0.5 | 70 | 1500 | 0.507 | 0.528 | 0.005 | 0.361 | 0.375 | 0.148 | 0.323 | 0.310 | 0.384 | 0.918 | 0.934 |
| -0.5 | 0.5 | 90 | 6000 | 0.492 | 0.503 | 0.012 | 0.333 | 0.276 | 0.095 | 0.302 | 0.152 | 0.308 | 0.908 | 0.936 |
| -0.5 | 0 | 50 | 6000 | 0.492 | 0.007 | 0.005 | 0.333 | 0.022 | 0.108 | 0.302 | 0.148 | 0.328 | 0.928 | 0.952 |
| -0.5 | 0 | 70 | 3000 | 0.512 | 0.005 | 0.000 | 0.347 | 0.041 | 0.104 | 0.293 | 0.201 | 0.322 | 0.940 | 0.934 |
| -0.5 | 0 | 90 | 1500 | 0.507 | 0.027 | 0.002 | 0.361 | 0.102 | 0.115 | 0.323 | 0.318 | 0.340 | 0.930 | 0.956 |
| 0.5 | -0.5 | 50 | 3000 | 0.488 | 0.515 | 0.022 | 0.324 | 0.309 | 0.128 | 0.293 | 0.209 | 0.357 | 0.948 | 0.944 |
| 0.5 | -0.5 | 70 | 1500 | 0.493 | 0.493 | 0.002 | 0.347 | 0.348 | 0.146 | 0.323 | 0.324 | 0.382 | 0.924 | 0.930 |
| 0.5 | -0.5 | 90 | 6000 | 0.508 | 0.506 | 0.009 | 0.349 | 0.279 | 0.094 | 0.302 | 0.153 | 0.307 | 0.908 | 0.958 |
| 0.5 | 0.5 | 50 | 6000 | 0.508 | 0.494 | 0.006 | 0.349 | 0.266 | 0.106 | 0.302 | 0.150 | 0.326 | 0.924 | 0.952 |
| 0.5 | 0.5 | 70 | 3000 | 0.488 | 0.503 | 0.001 | 0.324 | 0.294 | 0.104 | 0.293 | 0.200 | 0.322 | 0.938 | 0.936 |
| 0.5 | 0.5 | 90 | 1500 | 0.493 | 0.530 | 0.006 | 0.347 | 0.378 | 0.115 | 0.323 | 0.313 | 0.339 | 0.926 | 0.942 |
| 0.5 | 0 | 50 | 1500 | 0.493 | 0.012 | 0.003 | 0.347 | 0.089 | 0.187 | 0.323 | 0.298 | 0.432 | 0.938 | 0.934 |
| 0.5 | 0 | 70 | 6000 | 0.508 | 0.005 | 0.006 | 0.349 | 0.021 | 0.106 | 0.302 | 0.144 | 0.325 | 0.914 | 0.952 |
| 0.5 | 0 | 90 | 3000 | 0.488 | 0.004 | 0.01 | 0.324 | 0.043 | 0.091 | 0.293 | 0.207 | 0.302 | 0.93 | 0.950 |
| 0 | -0.5 | 50 | 6000 | 0.008 | 0.508 | 0.005 | 0.091 | 0.280 | 0.106 | 0.302 | 0.148 | 0.326 | 0.926 | 0.948 |
| 0 | -0.5 | 70 | 3000 | 0.012 | 0.507 | 0.000 | 0.086 | 0.300 | 0.103 | 0.293 | 0.207 | 0.321 | 0.944 | 0.938 |
| 0 | -0.5 | 90 | 1500 | 0.007 | 0.539 | 0.004 | 0.104 | 0.403 | 0.114 | 0.323 | 0.335 | 0.337 | 0.938 | 0.950 |
| 0 | 0.5 | 50 | 1500 | 0.007 | 0.514 | 0.006 | 0.104 | 0.352 | 0.185 | 0.323 | 0.295 | 0.431 | 0.938 | 0.932 |
| 0 | 0.5 | 70 | 6000 | 0.008 | 0.497 | 0.005 | 0.091 | 0.266 | 0.106 | 0.302 | 0.141 | 0.325 | 0.920 | 0.946 |
| 0 | 0.5 | 90 | 3000 | 0.012 | 0.497 | 0.007 | 0.086 | 0.286 | 0.090 | 0.293 | 0.196 | 0.300 | 0.926 | 0.946 |
| 0 | 0 | 50 | 3000 | 0.012 | 0.005 | 0.022 | 0.086 | 0.039 | 0.128 | 0.293 | 0.198 | 0.357 | 0.944 | 0.938 |
| 0 | 0 | 70 | 1500 | 0.007 | 0.026 | 0.006 | 0.104 | 0.094 | 0.147 | 0.323 | 0.306 | 0.384 | 0.922 | 0.926 |
| 0 | 0 | 90 | 6000 | 0.008 | 0.004 | 0.010 | 0.091 | 0.020 | 0.095 | 0.302 | 0.141 | 0.308 | 0.900 | 0.942 |
